# Outcomes and associated factors of revision procedures after failed total ankle arthroplasty: a comparative cohort analysis

**DOI:** 10.1007/s00402-026-06305-4

**Published:** 2026-04-17

**Authors:** Kathrin Pfahl, Julia Eder, Dominic Simon, Gautier Beckers, Boris Michael Holzapfel, Markus Walther

**Affiliations:** 1 Department of Orthopaedics and Trauma Surgery, Musculoskeletal University Center Munich (MUM), Ludwig-Maximilians-Universität München, Munich, Germany; 2 Center for Foot and Ankle Surgery, Schön Klinik München Harlaching, Munich, Germany; 3 Department of Psychiatry and Psychotherapy, Ludwig-Maximilians-Universität München, Munich, Germany; 4 Center for Foot and Ankle Surgery, Schön Klinik München Harlaching, Munich, Germany; 5 Department of Orthopedic Surgery, University of Würzburg, Wurzburg, Germany

**Keywords:** Prosthesis failure, Salvage procedure, Survivorship, Risk factor, Decision tree analysis

## Abstract

**Introduction:**

After failed total ankle arthroplasty (TAA), revision arthrodesis (RAA) and revision arthroplasty (RTAA) are treatment options, but comparative outcome data remain limited. The aim of this study was to compare mid-term survival of RAA and RTAA after failed primary TAA and to explore clinical and radiographic factors associated with failure.

**Materials and methods:**

In this retrospective cohort study, 124 patients (RAA = 72, RTAA = 52) were reviewed after failed TAA between 2006 and 2020, with a minimum follow-up of 12 months. Kaplan-Meier analysis and Cox regression were used to assess survival, and decision tree models were used as exploratory tools to identify factors associated with failure.

**Results:**

Mean follow-up was 71.6 ± 42.7 months. Surgical failure occurred in 6.45% of patients, with no significant difference (RAA 6.9%, RTAA 5.8%). Five-year survival was slightly higher for RTAA (97% vs. 93%), although durability decreased beyond 87 months. Female sex, higher BMI, and younger age were associated with failure after RAA, whereas large periprosthetic cysts and elevated BMI were related to failure after RTAA.

**Conclusions:**

RAA and RTAA demonstrate comparable mid-term survival after failed TAA. Revision strategy selection should be individualized, considering bone stock, patient characteristics, and failure patterns.

**Levels of evidence:**

III

## Introduction

Total ankle arthroplasty (TAA) continues to evolve as a treatment modality for end-stage ankle arthritis. Ten-year survivorship of modern TAA designs has been reported at approximately 89%, with some implant-specific series demonstrating survival rates exceeding 90% at long-term follow-up, although often accompanied by a substantial rate of additional procedures [1, 2]. More recent analyses have reported survival rates of up to 97.7% at 5-year follow-up for selected surgical techniques, such as transfibular TAA [3].

Long-term durability remains challenging, with revision rates of 12.6% at 7 years and 18% at 10 years [4, 5]. When TAA fails, salvage procedures include conversion to revision ankle arthrodesis (RAA) or revision total ankle arthroplasty (RTAA) [6, 7].

While primary ankle replacement and arthrodesis have been extensively studied, the literature on revision procedures is less robust. RAA has been associated with solid union rates (up to 90%), but carries risks of nonunion, pain, and adjacent joint degeneration [8–11]. RTAA outcomes vary widely, with 5-year survival rates ranging from 60% to 80% [12–15].

As patient comorbidities rise, it is vital to consider their impact on treatment outcomes [16].

Patient-related risk factors include elevated BMI (≥ 30 kg/m²), which has been linked to higher complication and nonunion rates after ankle arthrodesis, as well as an increased risk of wound problems and readmissions [17]. Elevated BMI has also been associated with an increased risk of prosthesis failure after primary total ankle arthroplasty [18, 19]. Age represents another relevant variable, as older patients may demonstrate impaired healing capacity and higher non-union rates [20, 21], whereas younger patients may place greater mechanical demands on the reconstruction [19, 22]. In addition, sex-related differences have been described, with female patients reported to experience inferior clinical outcomes and altered gait patterns after arthrodesis [21].

Implant- and pathology-related factors also influence outcomes. The presence of periarticular cysts has been associated with compromised bone stock and an increased risk of failure after TAA [18, 22]. Underlying diagnoses may further modify prognosis. Patients with rheumatoid arthritis have demonstrated superior TAA survivorship than those with osteoarthritis [19], whereas periprosthetic joint infections and infection-related periarticular ossifications are associated with higher failure rates [23–25].

Procedure-related risk factors, such as prior revision surgery, have been shown to result in less improvement after both TAA and ankle arthrodesis (AA) [16, 26].

Many comorbidities affect both primary TAA and AA similarly, complicating initial treatment decisions. Understanding these shared risk profiles is crucial for tailoring revision strategies once primary procedures fail.

However, there is a lack of comparative studies not only on survival but also on the impact of associated factors of failure, specifically in the context of RAA versus RTAA. Prior investigations have mainly focused on primary procedures.

To address these gaps, we aimed to: first, compare survival outcomes of RAA and RTAA following failed primary TAA; second, explore associated clinical and radiographic factors using decision tree analyses that were applied as exploratory tools to identify patterns within the dataset.

We hypothesized that survival may differ between RTAA and RAA following failed primary TAA, potentially reflecting advances in implant design. Additionally, we expected that various patient- and implant-related factors may be associated with revision failure.

## Methods

We included and retrospectively reviewed all patients who underwent either revision ankle arthrodesis (RAA) or revision total ankle arthroplasty (RTAA) for failed primary TAA at our center between 2006 and 2020. Cases with primary amputation or follow-up < 12 months were excluded. All 700 primary TAA patients during this period were included in the national implant registry maintained by the National Orthopedic Society. Follow-up visits and subsequent surgeries are likewise documented. Following ethics board approval, demographic characteristics, comorbidities, and reoperation rates were extracted from medical records, while radiographs and CT (computed tomography) scans were reviewed for radiographic assessment. Patient-reported outcome measures (PROMs) were collected.

A total of 124 revision cases met the inclusion criteria. The revision strategy aimed to preserve bone stock whenever possible. When sufficient bone was available, non-stemmed implants were used. In cases of tibial bone defects, stemmed implants were employed. Talar cysts or poor talar bone quality typically require revision implants with plate support. In the RAA group, 45 procedures involved isolated ankle arthrodesis, while 27 were conducted as tibiotalocalcaneal (TTC) fusions. Fusion technique depended on bone quality and deformity. Isolated ankle arthrodesis was predominantly performed using anterior plate fixation. In selected cases, lateral plating was used depending on deformity correction. TTC fusion was mostly stabilized with intramedullary nailing. Autologous bone grafting, most commonly harvested from the iliac crest, was used to address bone defects. In cases of extensive bone loss, a structural allograft was occasionally required. Among the RTAA cases, 21 were revised using a large-stemmed prosthesis and 31 using a resurfacing prosthesis.

We analyzed survivorship and potential associated factors for failure in both revision strategies. Variables considered included prior surgical procedures (e.g. ossification resection or wound debridement) or additional interventions (e.g. calcaneal osteotomy or subtalar fusion). “Time to revision” was defined as the interval in months from primary TAA to revision procedure. Further clinical factors included the underlying indication for RAA or RTAA, age, gender, BMI levels, and the presence of an underlying rheumatoid disease. Radiographic evaluation before revision surgery included the assessment of periprosthetic cysts and periarticular ossifications.

Regarding failure analysis, surgical and clinical failure were analyzed separately, as they represented distinct outcome dimensions.

Surgical failure of RAA was defined as the need for revision surgery, including re-revision arthrodesis due to nonunion, spacer implantation due to infection, or amputation. Surgical failure of RTAA was defined analogously as the need for revision surgery, including prosthesis explantation followed by revision arthrodesis, spacer implantation, or amputation. Surgical failure was classified as the event (‘death’) in the survival analysis.

Clinical failure was defined as a European Foot and Ankle Society (EFAS) score < 10 at follow-up. Questionnaires were sent to all included patients, resulting in a follow-up rate of 79.4%. Baseline characteristics of responders and non-responders were compared to assess potential response bias.

For survival analysis, only surgical failures were considered events. For decision tree analyses, both surgical and clinical failures were classified as failures.

**Fig. 1:**
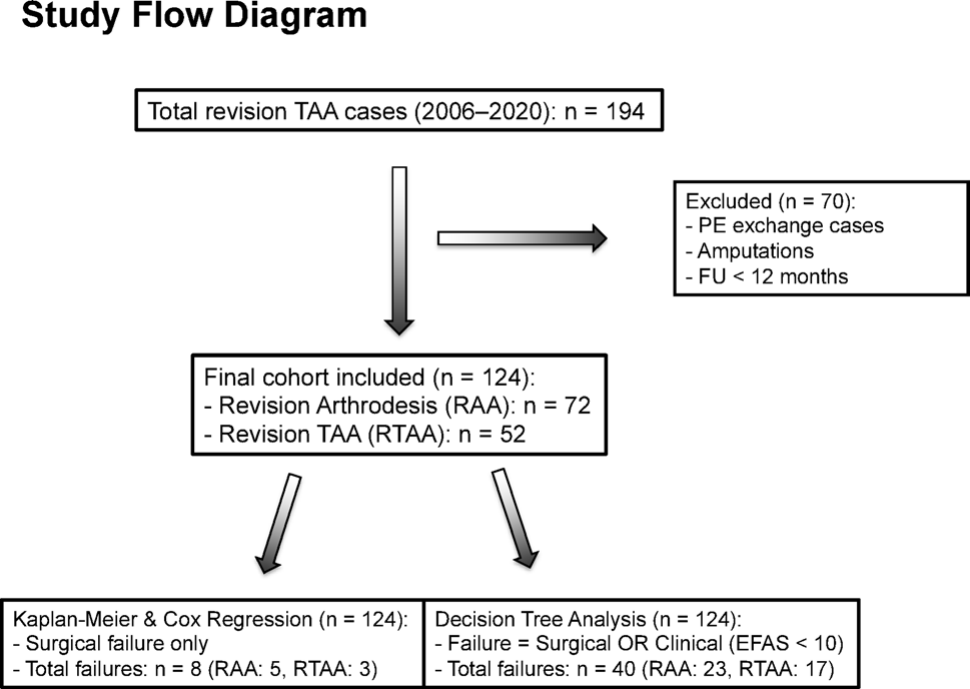
Study Flow Diagram (PE = Polyethylene exchange, FU = Follow up)

Figure 1 illustrates the composition of the study cohort and the distribution of surgical and clinical failures across the different analyses. Aseptic loosening, subsidence, and abrasion were summarized as component failure.

Descriptive statistics were used to analyze demographic and clinical data, failure rates, and clinical outcomes, and were expressed as means, standard deviations, percentages, and confidence intervals. We conducted group comparisons between RAA and RTAA using t-tests and chi-squared tests. For selected categorical predictors identified by the decision trees, odds ratios (OR) with 95% confidence intervals were calculated using contingency table analysis and Fisher’s exact test.

A survival analysis was performed using Kaplan–Meier curves to estimate the time to failure for each revision strategy. Failure was defined as the need for operative revision of the salvage procedure. The Cox proportional hazards model was applied to assess the effect of the type of revision procedure (RAA vs. RTAA) on hazard of failure. Hazard ratios (HRs) with corresponding 95% confidence intervals (CIs) and p-values were reported.

We implemented a 5-fold outer cross-validation loop to evaluate the model’s performance on unseen data. For each fold of the outer loop, the dataset was split into training and test sets.

An additional 3-fold inner cross-validation loop was used for hyperparameter tuning within the training set. Bootstrapping introduced variability by generating 50 resampled training datasets. Each bootstrap sample was used to train a decision tree classifier.

For each bootstrap sample, we conducted a grid search over a predefined hyperparameter space using GridSearchCV. Since the dataset was not balanced, with 32.26% of failures, the classes were weighted to achieve class balance.

The model with the highest validation accuracy was selected as the best model for the inner fold.

The best model from the inner loop was evaluated on the outer test set, and its accuracy was recorded.

This process was repeated for all outer folds, resulting in five accuracy scores, which were averaged to estimate the model’s generalization performance.

Given the exploratory and retrospective nature of this study, no a priori sample size calculation was performed. All statistical analyses were conducted using Microsoft Excel (Redmond, WA), JupyterLab Version 3.0.14 (Open-Source Project), and Python Version 3.9.16 (Beaverton, OR). The following packages were utilized for the study: Pandas (v1.5.2), SciPy (v1.10.0), NumPy (v1.24.2), Scikit-learn (v1.0.2), and Matplotlib (v3.6.2).

A p-value of ≤ 0.05 was considered statistically significant.

## Results

A total of 124 patients were included. Of these, 72 (58.06%) underwent RAA and 52 (41.94%) RTAA. Mean follow-up was 71.56 ± 42.66 months, with a minimum follow-up of 12 months. Mean age at revision was 63.28 ± 11.34 years, with 73 male (58.87%) and 51 female patients (41.13%). Average survival of the primary TAA was 77.95 ± 49.71 months.

The most frequently explanted primary implants were Salto Talaris (Integra, Plainsboro, NJ), Scandinavian Total Ankle Replacement (STAR; Stryker, Mahwah, NJ), and Hintegra (Artiqo, Luedinghausen). Revision implants for RTAA included Inbone II, Infinity (Wright Medical, Inc., Memphis, TN), and Salto XT (Integra, Plainsboro, NJ).

### Survival analysis and failures of salvage procedures

Surgical revision of the salvage procedure was defined as the event (‘death’) in survival analysis. A total of 8 salvage procedures (6.45%) failed during follow-up. In the RAA group, 5 cases (6.94%) required reoperation, whereas 3 (5.77%) failed in the RTAA group. One-year and five-year survival rates were 95% and 93% for RAA, and 98% and 97% for RTAA, respectively. After 87 months, the curves intersected, with the RTAA curve falling below that of RAA.

**Fig. 2:**
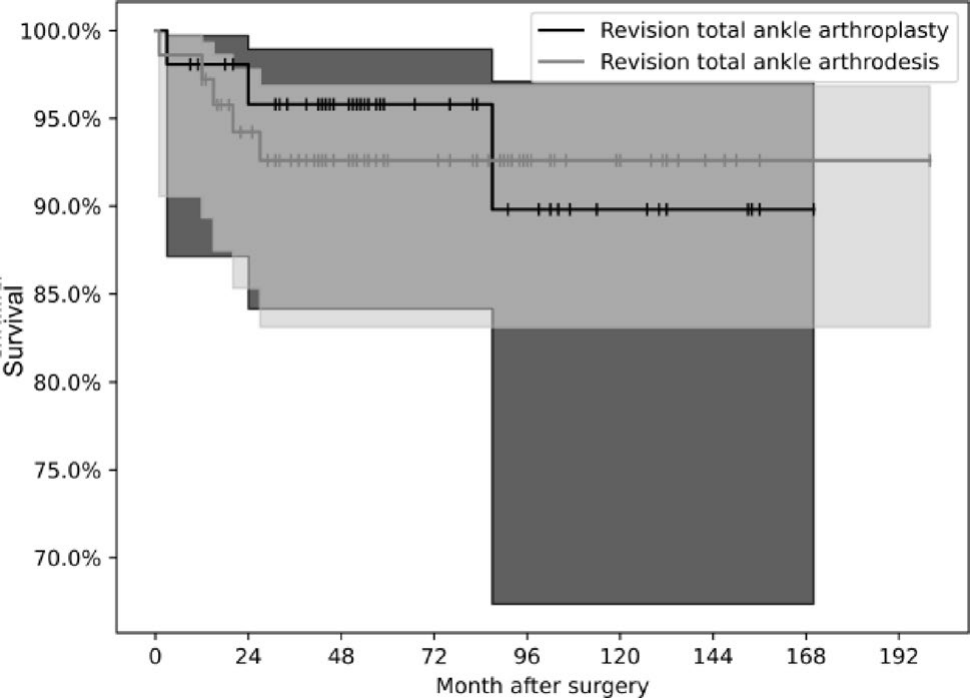
Kaplan-Meier-Curve of RAA and RTAA

Cox regression showed no significant difference between RAA and RTAA (b = -0.02, 95% CI: -0.38–0.34, HR = 0.98, *p* = 0.93). Kaplan-Meier survival curves (Fig. 2) demonstrate cumulative survival for each group. Confidence intervals widen beyond 7 years due to decreasing numbers at risk.

### Associated factors analysis

Group comparisons were performed to assess differences in potential factors associated with outcome between patients undergoing RAA and RTAA. Included factors, their distributions, and p-values are outlined in Table 1. Additional interventions were significantly more frequent in the RTAA group (*p* = 0.0007), whereas no other significant differences were observed.

**Table 1:** Associated factors, their distribution in the cohort, the RAA and RTAA group and p-values of group comparisons with the appropriate statistical analyses

Associated Factor	Cohort (*n* = 124)	Revision Arthrodesis (*n* = 72)	Revision Arthroplasty (*n* = 52)	*p*-value
Age in years M (SD)	63.28 ± 11.34	63.86 ± 12.21	62.48 ± 10.09	0.89 (t-test)
Sex				0.21 (Chi-squared test)
Male	73 (58.87%)	39 (54.17%)	34 (82.69%)	
*Female*	51 (41.13%)	33 (45.83%)	18 (34.62%)	
BMI in kg/m2 M (SD)	28.72 ± 4.7	28.96 ± 4.85	28.40 ± 4.51	0.06 (t-test)
Diagnosis				0.37 (Chi-squared test)
Component failure	90 (72.58%)	52 (72.22%)	38 (73.08%)	
Misalignment	22 (17.74%)	11 (15.28%)	11 (21.15%)	
PJI	12 (9.68%)	9 (12.5%)	3 (5.77%)	
Rheumatic Disease				0.81 (Chi-squared test)
None	101 (81.45%)	59 (81.94%)	42 (80.77%)	
Present,* but in remission*	3 (2.42%)	1 (1.39%)	2 (3.85%)	
Present and with antirheumatic treatment	6 (4.84%)	4 (5.56%)	2 (3.85%)	
Excluded	14 (11.29%)	8 (11.11%)	6 (11.54%)	
Previous Revisions				0.28 (Chi-squared test)
None	84 (67.74%)	46 (63.89%)	38 (73.08%)	
Present	40 (32.26%)	26 (26.11%)	14 (26.92%)	
Additional Procedures				**0.0007 (Chi-squared test)**
None	93 (75%)	62 (86.11%)	31 (59.62%)	
Present	31 (25%)	10 (13.89%)	21 (40.38%)	
Periprosthetic Cysts				0.45 (Chi-squared test)
None	26 (20.97%)	12 (16.67%)	14 (26.92%)	
present, but of size up to 1 cm	40 (32.26%)	23 (31.94%)	17 (32.69%)	
present and of size 1 cm or larger	44 (35.48%)	29 (40.28%)	15 (28.85%)	
Excluded	14 (11.92%)	8 (11.11%)	6 (11.54%)	
Periarticular Ossifications				0.61 (Chi-squared test)
None	31 (25%)	15 (20.83%)	16 (30.77%)	
Present but not spanning the joint space	56 (45.16%)	34 (47.22%)	22 (42.31%)	
Present and spanning the joint space	23 (18.55%)	15 (20.83%)	8 (15.38%)	
Excluded	14 (11.29%)	8 (11.11%)	6 (11.54%)	

### Decision tree analysis

For decision tree analyses failure definition was expanded to include both surgical and clinical failure. Clinical failure was defined by poor functional outcome, characterized by an EFAS score below 10. EFAS scores were available for 79.4% of patients (*n* = 98). Comparison between responders and non-responders revealed no significant differences regarding age, BMI, or revision procedure (all *p* > 0.05). However, a significant difference in sex distribution was observed (*p* = 0.02), with male patients being overrepresented among responders.

Thus, clinical failure occurred in 33 of 124 patients (26.61%). Nineteen patients (26.39%) in the RAA group and 14 patients (26.92%) in the RTAA group experienced clinical failure. Table 2 presents the distribution of surgical and clinical failures. Patients experiencing both surgical and clinical failure were counted once in the overall failure category (*n* = 1).

**Table 2:** Distribution of failures in the cohort, the RAA and RTAA group^a^

Failures	Cohort (*n* = 124)	Revision Arthrodesis (*n* = 72)	Revision Arthroplasty (*n* = 52)
Surgical Failures (due to necessary revision surgery)	8 (6.45%)	5 (6.94%)	3 (5.77%)
Clinical Failures (due to EFAS Score ≤ 10)	33 (26.61%)	19 (26.39%)	14 (26.92%)
Total Failures	40 (32.26%)	23 (31.94%)	17 (32.69%)

a One patient experienced both surgical and clinical failure and was counted once in the overall failure category

Separate decision tree analyses were performed for each salvage procedure to identify factors associated with failure

#### Decision tree for RAA

**Fig. 3:**
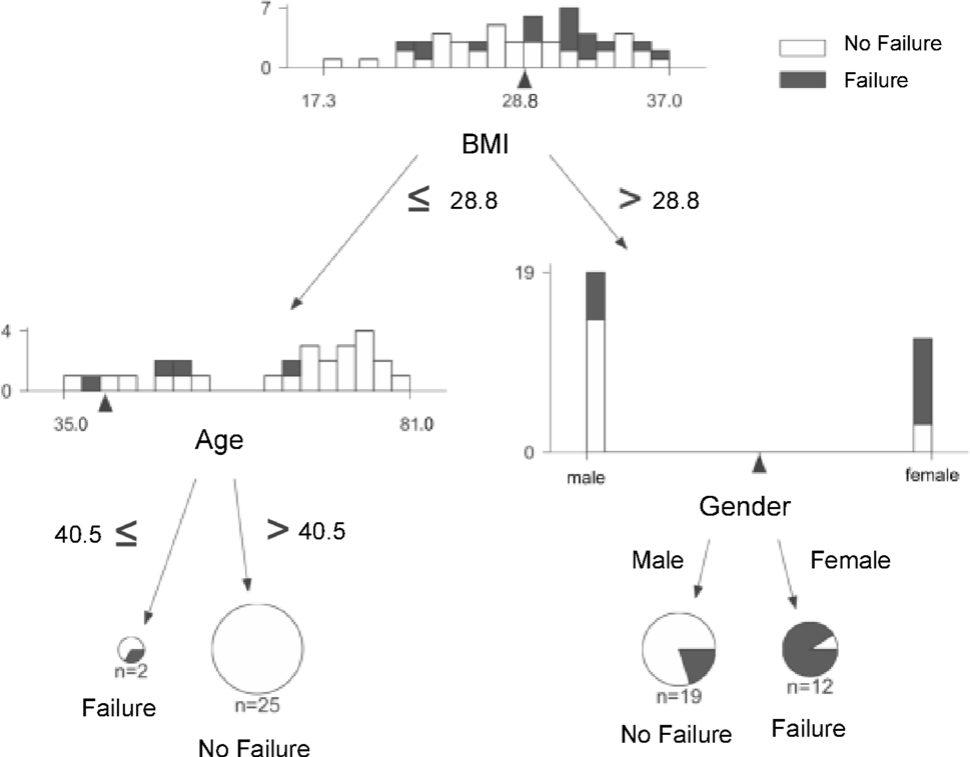
Decision tree analysis for revision arthrodesis (*n* = 72)

The decision tree for RAA identified female sex and BMI > 28.77 kg/m² as possible predictors of failure (Fig. 3).

Male patients with BMI ≤ 28.77 kg/m² showed a reduced risk. Of 23 total failures, 16 occurred in female patients and 7 in male patients. In univariate analyses, BMI > 28.77 kg/m² showed a strong association with failure (OR 8.94, 95% CI 2.62–30.54; *p* < 0.001), and female sex was associated with higher odds of failure after RAA (OR 4.30, 95% CI 1.48–12.48; *p* = 0.007). The data-driven age split at 40.5 years did not reach statistical significance in univariate comparison (OR 2.24, 95% CI 0.30–16.98; *p* = 0.436).

Most failures in female patients were related to poor clinical outcomes rather than surgical revision: 12 female patients (75%) exhibited low EFAS scores, while 4 (25%) required revision surgery.

Among patients with BMI ≤ 28.77 kg/m², age further modified risk: failure rates were lower in patients older than 40.5 years and higher in those younger than 40.5 years. The model showed good discriminative ability (AUC = 0.74, balanced accuracy 72.3%, sensitivity 64.0%, specificity 75.6%).

#### Decision tree for RTAA

**Fig. 4:**
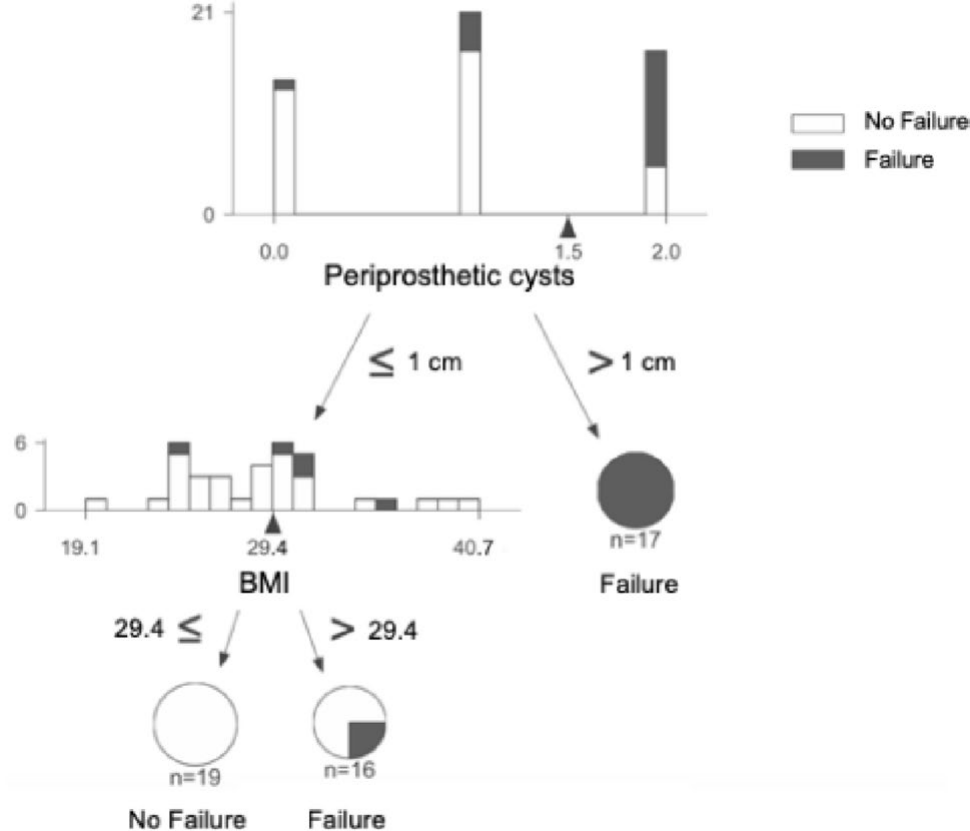
Decision tree analysis for revision arthroplasty (*n* = 52)

In the RTAA subgroup, periprosthetic cysts > 1 cm in diameter emerged as a primary associated factor for failure (Fig. 4).

Eleven of 15 patients (73%) with cysts > 1 cm failed, compared with 6 of 31 patients (19%) without such cysts. This corresponds to a relative risk of 3.79 and an odds ratio of 11.46 (95% CI: 2.63–49.9; *p* < 0.01).

When cysts were ≤ 1 cm and BMI was ≤ 29.37 kg/m², failure was less likely. However, failure rates increased significantly in the same scenario when BMI exceeded 29.37 kg/m². BMI > 29.37 kg/m² was associated with an increased odds of failure (OR 3.32, 95% CI 0.89–12.37; *p* = 0.07), although this did not reach statistical significance. The RTAA decision tree model exhibited robust performance (AUC = 0.64, balanced accuracy 64.52%, sensitivity 50.00%, specificity 77.14%).

## Discussion

We compared the survival and failure rates of revision ankle arthrodesis and revision total ankle arthroplasty following failed total ankle arthroplasty. By identifying associated factors that might influence the success or failure of these procedures, we aimed to support patient selection and surgical planning.

RTAA resulted in slightly superior survival rates beyond 5 years than RAA **(**97% vs. 93%**).** Earlier studies found overall union rates of around 80% for RAA and a four-year survival of 87% for RTAA [10].

Failures in this cohort were heterogeneous. Surgical failure (6.45%) accounted for a smaller proportion of events, whereas more failures were driven by poor clinical outcomes (26.61%) as reflected by low EFAS scores. Reported failure rates in the literature range from 6 to 27% for RAA [27–32] and 12–21% for RTAA [13, 14, 33–36]. A recent meta-analysis confirmed a five-year revision rate of 9.9% for RTAA [37].

In particular, the overall surgical failure rate was low in both groups, indicating that contemporary revision strategies after failed TAA can achieve reliable mid-term survivorship. Although a 4.35-fold higher failure rate for RAA compared to RTAA was observed in a previous study [38], our recent results with comparable survival curves for RAA and RTAA suggest that both procedures represent viable salvage options when appropriately selected. These findings support the notion that failure of a primary TAA does not necessarily preclude a successful revision outcome.

There was a significantly higher rate of additional procedures performed in the RTAA group compared to the RAA group. These interventions most commonly addressed residual deformity, compromised bone stock, or adjacent joint pathology. Previous reports indicated that successful RTAA often requires a more complex reconstructive strategy, including corrective osteotomies, subtalar procedures, or management of bone defects, compared to revision arthrodesis [12, 39, 40].

Despite the higher rate of additional interventions, RTAA did not demonstrate inferior mid-term survival in the present cohort, suggesting that the need for concomitant procedures should be interpreted as a marker of surgical complexity rather than an indicator of treatment failure.

Given the multifactorial nature of revision failure, treatment decisions should be individualized. The decision tree models were therefore applied as exploratory tools to illustrate potential interactions between variables within this dataset rather than to establish clinically validated decision algorithms.

In RAA, female sex, younger age, and higher BMI were linked to poorer outcomes. These findings may result from reduced bone healing capacity, hormonal differences, and comorbidities, which could contribute to poorer outcomes in female patients. Most failures in women were due to poor clinical outcomes rather than revisions, possibly related to difficulties adapting to altered gait mechanics and loss of ankle motion after arthrodesis. Prior research showed similar revision rates [41] but worse PROMs in women [42].

The association between younger age and poorer outcomes may be due to higher functional demands. Comparisons with preceding studies on primary AA illustrated that age was not predictive of revision after AA [41]. TAA was mainly recommended for older patients with low physical demands and AA for younger patients. Recent evidence, however, suggests that younger age (< 50 years) should not contraindicate TAA [43]. Our results extend this to revisions, supporting the notion that RTAA should also be considered for younger patients, as they might benefit from a better outcome. However, the identified age threshold of 40.5 years should be interpreted as a data-driven split within the decision tree rather than a strict clinical cutoff.

The influence of BMI is well-documented in AA, with morbid obesity (> 40 kg/m^2^) being associated with an increase in postoperative complications and in the odds of nonunion [44, 45]. Although some studies found no significant effect of obesity on RAA outcomes [26], our findings suggest that elevated BMI may still compromise RAA success.

RTAA was negatively affected by the presence of cysts larger than 1 cm and higher BMI values. Periprosthetic cysts have been associated with osteolysis, micromotion of the implant, wear particles, and synovial fluid pressure [18, 46]. They may indicate a higher degree of bone loss and structural instability, which could complicate fixation and compromise implant survival [47]. This finding is consistent with prior studies showing osteolysis worsens TAA revision outcomes [48].

Elevated BMI has been previously associated with higher rates of implant failure and complications following primary TAA [49], though newer data found no increased complication risk [50].

Regarding RTAA, elevated BMI levels have been associated with lower survival rates and higher revision rates of up to 17.8% [19, 21, 51].

These findings highlight distinct patterns associated with failure after RAA and RTAA. The decision tree analysis should be interpreted strictly as exploratory and hypothesis-generating. The models were internally validated and are not intended to serve as definitive clinical decision-making tools.

Several limitations to this study must be considered. Its retrospective nature introduces potential biases, and it is constrained by the relatively small number of patients and variations in patient variables. The follow-up duration was relatively limited for assessing long-term survivorship of revision procedures after failed TAA, and extended follow-up may reveal additional failures. The RAA group comprised both isolated ankle arthrodesis and TTC fusion procedures. These techniques differ in surgical complexity and biomechanical implications, potentially introducing heterogeneity within the RAA cohort. The study was not powered to perform a separate subgroup analysis for these fusion types, and results should therefore be interpreted with this limitation in mind. Furthermore, the overall sample size inherently limits the statistical power of the analysis. Given the rarity of revision procedures, the likelihood of a type II error must be considered, meaning that potentially relevant differences may not have reached statistical significance. In particular, the absolute number of surgical failure events was low, further limiting statistical robustness. No a priori power calculation was performed due to the retrospective design. Consequently, non–statistically significant differences between RAA and RTAA should not be interpreted as evidence of equivalence, but rather as findings requiring cautious interpretation and confirmation in larger cohorts.

EFAS scores were available for 79.4% of patients. Although no significant differences were observed regarding age, BMI, or revision type between responders and non-responders, the sex distribution differed significantly. Therefore, a potential response bias cannot be fully excluded. Defining clinical failure using the EFAS score required a pragmatic cut-off below the cohort average EFAS score of 12.31. The threshold also needed to account for patients’ increasing demands on their daily and leisure activities. If a different cut-off point had been chosen, it could have yielded different outcomes. The EFAS score was only assessed postoperatively. It cannot be determined whether patients already had low scores preoperatively before undergoing primary TAA. Therefore, the clinical failure rate cannot be interpreted in isolation, as it doesn’t consider the patients’ baseline condition prior to primary TAA. This limits the interpretation of our findings, as some cases with postoperative EFAS < 10 may not reflect a true clinical failure. For future studies, it would be highly valuable to collect and compare PROMs both preoperatively and postoperatively.

Decision trees are biased by features with many levels, potentially neglecting features with fewer levels. They tend to be unstable due to their sensitivity to data variations, leading to different decision boundaries and predictions given slight changes in the data set.

Considering the small number of cases, our findings must therefore be interpreted with caution. It is crucial to replicate our findings across larger data sets to validate the generalizability of the decision tree models presented [52]. However, we could assess the generalizability of the models by the internal validation dataset, which was split off before training the decision trees.

### Conclusion

In this study, both RAA and RTAA achieved high mid-term survival rates, suggesting that failed primary TAAs can be effectively revised. With slightly better survival rates for RTAA than for RAA, neither procedure is universally superior. Additional interventions were performed more frequently in the RTAA group, which might reflect the higher technical complexity often required in revision arthroplasty. However, both options are influenced by specific risk profiles. By integrating factors associated with failure into decision tree models, we used an exploratory approach to illustrate patterns associated with failure and to demonstrate how multiple factors may interact across different revision strategies. RTAA may be considered when bone stock is adequate, whereas RAA may remain a suitable option, particularly in elderly, male patients with bone loss. Future prospective studies with larger sample sizes are needed to confirm these associations and ensure broader clinical applicability.

## Data Availability

No datasets were generated or analysed during the current study.

## References

[1] Zaidi R, Cro S, Gurusamy K, Siva N, Macgregor A, Henricson A et al (2013) The outcome of total ankle replacement: a systematic review and meta-analysis. Bone Joint J 95–b(11):1500–1507. 10.1302/0301-620x.95b11.31633 24151270 10.1302/0301-620X.95B11.31633

[2] Jastifer JR, Coughlin MJ (2015) Long-term follow-up of mobile bearing total ankle arthroplasty in the United States. Foot Ankle Int 36(2):143–150. 10.1177/1071100714550654 25201330 10.1177/1071100714550654

[3] Maccario C, Paoli T, Romano F, D’Ambrosi R, Indino C, Usuelli FG (2022) Transfibular total ankle arthroplasty: a new reliable procedure at five-year follow-up. Bone Joint J 4472–478. 10.1302/0301-620x.104b4.Bjj-2021-0167.R5. 104-b 10.1302/0301-620X.104B4.BJJ-2021-0167.R5 35360940

[4] Gougoulias N, Khanna A, Maffulli N (2010) How successful are current ankle replacements? a systematic review of the literature. Clin Orthop Relat Res 468(1):199–208. 10.1007/s11999-009-0987-3 19618248 10.1007/s11999-009-0987-3 PMC2795846

[5] Hauer G, Hofer R, Kessler M, Lewis J, Leitner L, Radl R et al Revision Rates After Total Ankle Replacement: A Comparison of Clinical Studies and Arthroplasty Registers. Foot Ankle Int 2021 Nov 12:10711007211053862. 10.1177/10711007211053862 10.1177/10711007211053862 34766517

[6] Kotnis R, Pasapula C, Anwar F, Cooke PH, Sharp RJ (2006) The management of failed ankle replacement. J Bone Joint Surg Br 88(8):1039–1047. 10.1302/0301-620x.88b8.16768 16877603 10.1302/0301-620X.88B8.16768

[7] Jennison T, Spolton-Dean C, Rottenburg H, Ukoumunne O, Sharpe I, Goldberg A (2022) The outcomes of revision surgery for a failed ankle arthroplasty: a systematic review and meta-analysis. Bone Jt Open 3(7):596–606. 10.1302/2633-1462.37.Bjo-2022-0038.R1 35880516 10.1302/2633-1462.37.BJO-2022-0038.R1 PMC9350690

[8] Gross C, Erickson BJ, Adams SB, Parekh SG (2015) Ankle arthrodesis after failed total ankle replacement: a systematic review of the literature. Foot Ankle Spec 8(2):143–151. 10.1177/1938640014565046 25561701 10.1177/1938640014565046

[9] Kamrad I, Henricson A, Magnusson H, Carlsson Å, Rosengren BE (2016) Outcome After Salvage Arthrodesis for Failed Total Ankle Replacement. Foot Ankle Int 37(3):255–261. 10.1177/1071100715617508 26582180 10.1177/1071100715617508

[10] Egglestone A, Kakwani R, Aradhyula M, Kingman A, Townshend D (2020) Outcomes of revision surgery for failed total ankle replacement: revision arthroplasty versus arthrodesis. Int Orthop 44(12):2727–2734. 10.1007/s00264-020-04784-7 32875387 10.1007/s00264-020-04784-7

[11] Jennison T, Ukoumunne OC, Lamb S, Sharpe I, Goldberg AJ (2023) Fusion after a failed primary total ankle arthroplasty. Bone Joint J 10105–b. 10.1302/0301-620x.105b10.Bjj-2023-0010.R1 10.1302/0301-620X.105B10.BJJ-2023-0010.R1 37777204

[12] Jamjoom BA, Siddiqui BM, Salem H, Raglan M, Dhar S (2022) Clinical and Radiographic Outcomes of Revision Total Ankle Arthroplasty Using the INBONE II Prosthesis. J Bone Joint Surg Am 104(17):1554–1562. 10.2106/jbjs.21.01240 35766416 10.2106/JBJS.21.01240

[13] Jennison T, Ukoumunne OC, Lamb S, Goldberg AJ, Sharpe I (2023) Survival of revision ankle arthroplasty. Bone Joint J 11105–b. 10.1302/0301-620x.105b11.Bjj-2023-0199.R1 10.1302/0301-620X.105B11.BJJ-2023-0199.R1 37909151

[14] Hintermann B, Peterhans US, Susdorf R, Horn Lang T, Ruiz R, Kvarda P (2024) Survival and risk assessment in revision arthroplasty of the ankle. Bone Joint J 106–b(1):46–52. 10.1302/0301-620x.106b1.Bjj-2023-0716.R2 38160692 10.1302/0301-620X.106B1.BJJ-2023-0716.R2

[15] Sundet M, Gyllensten KS, Dybvik E, Eikvar KH, Hallan G, Lillegraven S et al (2024) Five-Year Results of the Salto XT Revision Ankle Arthroplasty. Foot Ankle Int 45(10):1083–1092. 10.1177/10711007241264561 39075764 10.1177/10711007241264561 PMC11529115

[16] Cunningham DJ, DeOrio JK, Nunley JA, Easley ME, Adams SB (2019) The Effect of Patient Characteristics on 1 to 2-Year and Minimum 5-Year Outcomes After Total Ankle Arthroplasty. J Bone Joint Surg Am 101(3):199–208. 10.2106/jbjs.18.00313 30730479 10.2106/JBJS.18.00313

[17] Kuttner NP, Owen AR, Ryssman DB, Kitaoka HB, Turner NS (2025) Association of Complication Rates and Severe Obesity in Patients Undergoing Ankle Arthrodesis. Foot Ankle Int 46(2):210–216. 10.1177/10711007241300327 39611425 10.1177/10711007241300327

[18] Espinosa N, Klammer G, Wirth SH (2017) Osteolysis in Total Ankle Replacement: How Does It Work? Foot Ankle Clin 22(2):267–275. 10.1016/j.fcl.2017.01.001 28502348 10.1016/j.fcl.2017.01.001

[19] Jennison T, Ukoumunne OC, Lamb S, Sharpe I, Goldberg A (2023) Risk Factors for Failure of Total Ankle Replacements: A Data Linkage Study Using the National Joint Registry and NHS Digital. Foot Ankle Int 44(7):596–603. 10.1177/10711007231176512 37345846 10.1177/10711007231176512

[20] Berlet GC, Baumhauer JF, Glazebrook M, Haddad SL, Younger A, Quiton JD et al (2020 Oct-Dec) The Impact of Patient Age on Foot and Ankle Arthrodesis Supplemented with Autograft or an Autograft Alternative (rhPDGF-BB/β-TCP). 5(4). 10.2106/jbjs.Oa.20.00056. JB JS Open Access 10.2106/JBJS.OA.20.00056 PMC7757837 33376929

[21] Suh DH, Han K, Lee JW, Kim HJ, Kim B, Koo BM et al (2021) Risk factors associated with failure of total ankle arthroplasty: a nationwide cohort study. Sci Rep 11(1):2878. 10.1038/s41598-021-82674-7 33536553 10.1038/s41598-021-82674-7 PMC7859193

[22] Hanna RS, Haddad SL, Lazarus ML (2007) Evaluation of periprosthetic lucency after total ankle arthroplasty: helical CT versus conventional radiography. Foot Ankle Int 28(8):921–926. 10.3113/fai.2007.0921 17697658 10.3113/FAI.2007.0921

[23] Manrique J, Alijanipour P, Heller S, Dove M, Parvizi J (2018) Increased Risk of Heterotopic Ossification Following Revision Hip Arthroplasty for Periprosthetic Joint Infection. Arch Bone Jt Surg 6(6):486–491 30637303 PMC6310194

[24] Ohlmeier M, Krenn V, Thiesen DM, Sandiford NA, Gehrke T, Citak M (2019) Heterotopic Ossification in Orthopaedic and Trauma surgery: A Histopathological Ossification Score. Sci Rep 9(1):18401. 10.1038/s41598-019-54986-2 31804584 10.1038/s41598-019-54986-2 PMC6895226

[25] Pfahl K, Röser A, Gottschalk O, Hörterer H, Mehlhorn A, Dolp PA et al (2022) Common bacteria and treatment options for the acute and chronic infection of the total ankle arthroplasty. Foot Ankle Surg 28(7):1008–1013. 10.1016/j.fas.2022.02.010 35210186 10.1016/j.fas.2022.02.010

[26] O’Connor KM, Johnson JE, McCormick JJ, Klein SE (2016) Clinical and Operative Factors Related to Successful Revision Arthrodesis in the Foot and Ankle. Foot Ankle Int 37(8):809–815. 10.1177/1071100716642845 27044542 10.1177/1071100716642845

[27] Kitaoka HB, Romness DW (1992) Arthrodesis for failed ankle arthroplasty. J Arthroplasty 7(3):277–. 10.1016/0883-5403(92)90049-v. 84 1402943 10.1016/0883-5403(92)90049-v

[28] Hopgood P, Kumar R, Wood PL (2006) Ankle arthrodesis for failed total ankle replacement. J Bone Joint Surg Br 88(8):1032–1038. 10.1302/0301-620x.88b8.17627 16877602 10.1302/0301-620X.88B8.17627

[29] Culpan P, Le Strat V, Piriou P, Judet T (2007) Arthrodesis after failed total ankle replacement. J Bone Joint Surg Br 89(9):1178–1183. 10.1302/0301-620x.89b9.19108 17905954 10.1302/0301-620X.89B9.19108

[30] Thomason K, Eyres KS (2008) A technique of fusion for failed total replacement of the ankle: tibio-allograft-calcaneal fusion with a locked retrograde intramedullary nail. J Bone Joint Surg Br 90(7):885–888. 10.1302/0301-620x.90b7.20221 18591597 10.1302/0301-620X.90B7.20221

[31] Berkowitz MJ, Clare MP, Walling AK, Sanders R (2011) Salvage of failed total ankle arthroplasty with fusion using structural allograft and internal fixation. Foot Ankle Int 32(5):S493–502. 10.3113/fai.2011.0493 21733457 10.3113/FAI.2011.0493

[32] Wu KA, Anastasio AT, DeOrio JK, Nunley JA, Easley ME, Adams SB (2025) Exploring Revision Total Ankle Arthroplasty Failures: A Comparison Between Failed and Successful Revision Cases. Foot Ankle Spec 18(3):286–294. 10.1177/19386400241274551 39305052 10.1177/19386400241274551

[33] Ellington JK, Gupta S, Myerson MS (2013) Management of failures of total ankle replacement with the agility total ankle arthroplasty. J Bone Joint Surg Am 95(23):2112–2118. 10.2106/jbjs.K.00920 24306698 10.2106/JBJS.K.00920

[34] Hintermann B, Zwicky L, Knupp M, Henninger HB, Barg A (2013) HINTEGRA revision arthroplasty for failed total ankle prostheses. J Bone Joint Surg Am 95(13):1166–1174. 10.2106/jbjs.L.00538 23824384 10.2106/JBJS.L.00538

[35] Lachman JR, Ramos JA, Adams SB, Nunley JA, Easley ME, DeOrio JK (2019) Revision Surgery for Metal Component Failure in Total Ankle Arthroplasty. Foot Ankle Orthop 4(1):2473011418813026. 10.1177/2473011418813026 35097311 10.1177/2473011418813026 PMC8500383

[36] Lee JW, Im WY, Song SY, Choi JY, Kim SJ (2021) Analysis of early failure rate and its risk factor with 2157 total ankle replacements. Sci Rep 11(1):1901. 10.1038/s41598-021-81576-y 33479348 10.1038/s41598-021-81576-y PMC7820457

[37] Sun N, Li H, Li X, Li H, Lai L, Wu Y et al (2025) Fate of revision total ankle arthroplasty: a meta-analysis of 999 cases. Int J Surg 111(5):3561–3572. 10.1097/js9.0000000000002340 40101126 10.1097/JS9.0000000000002340 PMC12165482

[38] Pfahl K, Röser A, Eder J, Gottschalk O, Hörterer H, Mehlhorn A et al (2023) Outcomes of Salvage Procedures for Failed Total Ankle Arthroplasty. Foot Ankle Int 44(4):262–269. 10.1177/10711007231156426 36879477 10.1177/10711007231156426

[39] Horisberger M, Henninger HB, Valderrabano V, Barg A (2015) Bone augmentation for revision total ankle arthroplasty with large bone defects. Acta Orthop 86(4):412–414. 10.3109/17453674.2015.1009673 25619728 10.3109/17453674.2015.1009673 PMC4513594

[40] Wu KA, Anastasio AT, Wu JA, Ralph J, Jing C, Krez AN et al (2025) Indications and outcomes of revision total ankle arthroplasty. Expert Rev Med Devices 22(7):725–737. 10.1080/17434440.2025.2509770 40394854 10.1080/17434440.2025.2509770

[41] Randsborg PH, Jiang H, Mao J, Devlin V, Marinac-Dabic D, Peat R et al (2022) Apr-Jun Two-Year Revision Rates in Total Ankle Replacement Versus Ankle Arthrodesis: A Population-Based Propensity-Score-Matched Comparison from New York State and California. JB JS Open Access. ;7(2)10.2106/jbjs.Oa.21.00136 10.2106/JBJS.OA.21.00136 PMC9484817 36147655

[42] Dodd A, Pinsker E, Younger ASE, Penner MJ, Wing KJ, Dryden PJ et al (2022) Sex Differences in End-Stage Ankle Arthritis and Following Total Ankle Replacement or Ankle Arthrodesis. J Bone Joint Surg Am 104(3):221–228. 10.2106/jbjs.21.00287 35007215 10.2106/JBJS.21.00287

[43] Samaila EM, Bissoli A, Argentini E, Negri S, Colò G, Magnan B (2020) Total ankle replacement in young patients. Acta Biomed 91(4–s):31–35. 10.23750/abm.v91i4-S.9725 32555074 10.23750/abm.v91i4-S.9725 PMC7944830

[44] Kamalapathy PN, Du Plessis MI, Chen D, Bell J, Park JS, Werner BC (2021 Nov-Dec) Obesity and Postoperative Complications Following Ankle Arthrodesis: A Propensity Score Matched Analysis. J Foot Ankle Surg 60(6):1193–1197. 10.1053/j.jfas.2021.05.004 10.1053/j.jfas.2021.05.004 34127372

[45] Wong LH, Chrea B, Meeker JE, Yoo JU, Atwater LC (2022) Factors Associated With Nonunion and Infection Following Ankle Arthrodesis Using a Large Claims Database: Who Has Elevated Risk? Foot Ankle Orthop 7(2):24730114221101617. 10.1177/24730114221101617 35662901 10.1177/24730114221101617 PMC9158424

[46] Gaden MT, Ollivere BJ (2013) Periprosthetic aseptic osteolysis in total ankle replacement: cause and management. Clin Podiatr Med Surg 30(2):145–155. 10.1016/j.cpm.2012.10.006 23465805 10.1016/j.cpm.2012.10.006

[47] Lee GW, Seo HY, Jung DM, Lee KB (2021) Comparison of Preoperative Bone Density in Patients With and Without Periprosthetic Osteolysis Following Total Ankle Arthroplasty. Foot Ankle Int 42(5):575–581. 10.1177/1071100720976096 33349052 10.1177/1071100720976096

[48] Lee GW, Lee KB (2022) Periprosthetic Osteolysis as a Risk Factor for Revision After Total Ankle Arthroplasty: A Single-Center Experience of 250 Consecutive Cases. J Bone Joint Surg Am 104(15):1334–1340. 10.2106/jbjs.21.01093 35930380 10.2106/JBJS.21.01093

[49] Hermus JPS, van Kuijk SMJ, Spekenbrink-Spooren A, Witlox MA, Poeze M, van Rhijn LW et al (2022) Risk factors for total ankle arthroplasty failure: A Dutch Arthroplasty Register study. Foot Ankle Surg 28(7):883–886. 10.1016/j.fas.2021.12.001 34949541 10.1016/j.fas.2021.12.001

[50] Kim BI, Anastasio AT, Wixted CM, DeOrio JK, Nunley JA 2nd, Easley ME et al (2023) Total Ankle Arthroplasty: Does Obes Matter? Foot Ankle Int 44(7):587–595. 10.1177/10711007231171084 10.1177/10711007231171084 37345836

[51] Sansosti LE, Van JC, Meyr AJ (2018 Mar-Apr) Effect of Obesity on Total Ankle Arthroplasty: A Systematic Review of Postoperative Complications Requiring Surgical Revision. J Foot Ankle Surg 57(2):353–356. 10.1053/j.jfas.2017.10.034 10.1053/j.jfas.2017.10.034 29284576

[52] Podgorelec V, Kokol P, Stiglic B, Rozman I (2002) Decision trees: an overview and their use in medicine. J Med Syst 26(5):445–463. 10.1023/a:1016409317640 12182209 10.1023/a:1016409317640

